# Medical Flag over Alcoholic Mixtures

**Published:** 1886-10

**Authors:** 


### MEDICINAL FLAG OVER ALCOHOLIC MIXTURES.

The circular letter of Collector T. C. Crenshaw to deputy collectors, giving them instructions about certain means now being used to evade the prohibition law, by placing upon the market certain preparations of alcohol as medicines, states that no special tax will be required for compounding and preparing, or for selling medicines and medicinal preparations and compounds, which are really such in character as well as in name. But persons engaged in the manufacture of such compounds, under the name of medicines, should be regarded as rectifiers if the compounds are suitable for and are, when sold, used as alcoholic beverages, and those who thus sell them will be liable to pay the special tax.

The whole bearing of this subterfuge is thus clearly set forth in the accompanying explanatory remarks of the collector, and no one can plead ignorance of the proper construction of the law in violating it, nor should they receive any sympathy in being subjected to the penalties of such violation. We trust there are no reputable druggists in Atlanta who will undertake such evasions of law, since they are allowed to furnish all medicinal preparations with alcoholic ingredients that may be required by the sick, and the medical profession cannot prescribe them for others than the sick without a compromise of honor and good faith.

### ITEMS.

ATLANTA has an oculist who collects $2,000 a month from his practice.

DR. N. O. HARRIS has recently been elected City Physician of the Sixth Ward.

PROF. HUNTER P. COOPER will deliver the opening address at the Atlanta Medical College on Thursday, October 7th, at 11 o'clock a. m.

DR. R. O. COTTER, of Macon, passed through Atlanta a few days ago on his return from New York, where he has been for several months.

DR. J. C. AVARY, of this city, has moved to Charlotte, North Carolina. We regret to lose Dr. Avary. The best wishes of THE JOURNAL go with him to his new home.

DR. PAUL FAVER, of Fayetteville, a prominent member of the Medical Association of Georgia, was in Atlanta a few days since and gave THE JOURNAL a pleasant call.

THE Southern Homœopathic Medical Association will hold its third annual meeting at New Orleans, La., on Wednesday, Thursday and Friday, December 8, 9 and 10, 1886.

A CASE OF NEPHROTOMY.—Dr. J. McF. Gaston, of this city, recently made a nephrotomy for abscess, caused by calculi in the ureter. Two small stones were found, which had completely blocked up the ureter, preventing anything from passing into the bladder.

WE are in receipt of a programme of the exercises of the fourth annual meeting of the American Rhinological Association, to be held in St. Louis, October 6, 7 and 8, 1886. Dr. A. G. Hobbs, of this city, will lead in the discussion on "Scarification in Nasal Hypertrophy" on the morning of the second day.

A HOSPITAL FOR ATLANTA.—Our editorial in the September number of THE JOURNAL, calling attention to the necessity of a hospital in Atlanta, has awakened interest in the matter. Dr. Earnest read a paper before the Atlanta Society of Medicine on the subject at the last meeting. After free discussion a resolution was adopted calling a meeting of the profession of the city at an early day to discuss the question of the establishment of a hospital in Atlanta.

THE APPLICATION OF INTRA-UTERINE PRESSURE BY THE TAMPON.—We desire to call attention to an interesting article in this issue of THE JOURNAL from the pen of Dr. V. H. Taliaferro, of this city. This article was promised by Dr. Taliaferro in a communication to THE JOURNAL in July last for our August number, but has been delayed until now on account of the sickness of the doctor.

THE ECLECTIC.—The October *Eclectic*, published by E. R. Pelton, 25 Bond street, New York, has been laid before us, and offers great attractions to its readers. Sir John Lubbock has the place of honor in a disquisition on the "Study of Science," and this is well supported in the next paper, on "Pasteur and Hydrophobia," by Prof. Ray Lankester. One of the greatest men ever produced in America, Alexander Hamilton, is discussed by A. G. Bradley, and the well-known critic, George Saintsbury, has something to say about one of the Scottish intellectual giants, Christopher North, the founder of *Blackwood's Magazine.* Goldwin Smith's paper on the "Capital of the United States" will be read with interest. Other leading papers are Alex. H. Japp's "Some Unconscious Confessions of DeQuincey," and a very readable paper by Sophie Weisse on the great German historian, Ranke, with reminiscences of Berlin from 1884 to 1886. There are a number of other interesting papers by well known writers.

HYDRONAPHTHOL.—Dr. Justus Wolff asserts that E. Mercks' statement that betanaphthol and hydronaphthol are identical is a mistake, which may result in the most serious consequences if betanaphthol be used instead of hydronaphthol, "as the first one is a most dangerous and deadly poison, whilst the latter is an excellent, absolutely reliable and harmless antiseptic." The poisonous character of betanaphthol has been established a long time ago by such authorities as Kaposi, Neisser and Piffard, and lately by Max Schwarz, while Dr. G. R. Fowler, Dr. Lawrence Wolff and many others have proved hydronaphthol to be non-poisonous and a most effective antiseptic. Hydronaphthol is distinguished from betanaphthol, not only by its physiological action, but also by distinct chemical reactions and by its chemical constitution, as it possesses certainly more hydrogen in the molecular than betanaphthol. Of the several distinguishing chemical reactions, the following may be given as an example: If from a diluted iron-perchloride solution two drops are added to an alcoholic betanaphthol solution it becomes of a bright green color, whilst the same proportion of an alcoholic hydronaphthol solution of the same strength becomes dark yellowish brown by addition of the same proportion of iron-perchloride solution. Other reactions are also different, and the melting points obtained by most careful determinations are for hydronaphthol 117° C. and for betanaphthol 122° C. These and other facts satisfy the author that hydronaphthol is distinct from the poisonous compound which is known as betanaphthol, and that it is not alphanaphthol nor a mixture of the two last named and does not contain any of either.—*Druggists' Circular, September, 1886.*

A RARE FRACTURE OF THE ULNA.—Dr. C. B. Jackson, Notasulga, Ala., writes: "A child two years old fell from a wagon in motion, and fractured the ulna about three-fourths of an inch above the lower extremity. The inferior fragment was projecting on the dorsal surface, and the hand was deflected to the ulna side, producing a marked deformity. Crepitation was distinct. I applied a palmer and dorsal splint with graduated compress, and by means of adhesive plaster deflected the hand slightly to the radial side. The splints were removed on the eighteenth day, when union was found to be perfect. The only question with regard to which I am not satisfied is the cause of the fracture. There was no appreciable contusion, nor other evidence of direct force, and had the child caught its weight on the hand it certainly would have fractured the radius instead of the ulna."

THE CENTURY FOR 1886–7.—The leading feature for *The Century* for 1886-7 will be the Authorized Life of Lincoln, by his confidential Secretaries, John George Nicolay (now Marshal of the Supreme Court of the United States) and Col. John Hay (lately Assistant Secretary of State of the United States). This work, which was begun with the sanction and assistance of President Lincoln himself, and has been continued under the authority of the sole survivor of the President's immediate family, has been in active preparation during the past sixteen years. It is the only full and authoritative record of the private life and public career of

Abraham Lincoln, including an account of the causes of the rebellion, and a record, at first hand, of the inside history of the civil war, and of President Lincoln's administration, important details of which last have hitherto remained unrevealed, in order that they might first appear in their proper connection in this authentic history.

DR. T. M. MCINTOSH, of Thomasville, passed through Atlanta a few days since on his way to New York.

A TYPOGRAPHICAL error occurs in the head-line of Dr. Holmes' paper, on page 493, which we regret very much.

PAPINE.—Dr. Wm. J. Crittenden, of Unionville, Va. (*Virginia Medical Monthly*, August, 1886), says of this preparation: During January, 1886, I was called to see a lady suffering with acute peritonitis. She assured me that she could not use opium, as she had tired of it previously. But I gave her one-eighth grain of morphia sulphate and one one-hundred-and-twentieth grain of atropia sulphate hypodermically, and in a few minutes the depressing effects were noted, both upon the respiration and circulation; the pupils also became visibly contracted. I then tried the various usual substitutes for morphia in succession, but to no effect. I determined to try papine, but not being able to give it by the mouth on account of nausea, and as she objected to the use of the hypodermic needle, I gave her two drachms per rectum and repeated it in one hour. The result was that she sank into a quiet, peaceful sleep, which lasted for several hours. During the remainder of her sickness, I gave her papine with the most gratifying results. As soon as her stomach would retain it, I gave it to her by the mouth in one-drachm doses. I have also used papine in a case of uterine cancer in lieu of morphia. In cases which patients have been taking morphia until it has lost its anodyne influence papine is well adapted.

REFLEX NERVOUS PHENOMENA FROM INJURIES TO THE TESTICLE.—We note the two following cases illustrating serious reflex symptoms following wounds of the testicle and the tunica vaginalis. The first occurred during the tapping of a hydrocele, and is described in the *Lancet*, July, 1886, p. 23. M. Loumeau tapped a hydrocele in a healthy man, aged forty-four, and drew off about one hundred and twenty-five grammes of clear fluid. The operator then injected gently sixty grammes of a mixture of tincture of iodine with twice its volume of water. All at once the patient complained of severe pain in the spermatic cord and loins, with cramp in the right forearm. The ulnar border of the right hand then became flexed, the ring and small fingers being completely bent, while the index and middle fingers became flexed at their metacarpo-phalangeal articulations. The thumb also was flexed and brought near the fingers. Exactly the same position was shortly afterwards assumed by the left hand. There were no convulsions nor syncope. After a few minutes the ulnar muscles began to relax, and the index and middle fingers became flexed completely on the hand, which itself became strongly flexed on the forearm. All the muscles on the front of the forearm became hard and contracted. The palmar fascia was strongly retracted, and the palmaris brevis quite tense. On both sides the ulnar affection had given place to contraction of the muscles supplied by the median nerve. The tongue hung loosely in the mouth, and the patient was unable to articulate a sound. The forearms were shampooed, and nearly an hour afterwards the muscles relaxed. The patient recovered completely, leaving the hospital cured, in a few days. The author had no idea of the cause of this attack, but attributed it to reflex irritation of the nerves of the serous membrane of the testicle.—*Boston Medical and Surgical Journal.*

CONDITION OF A BODY AFTER DEATH FROM LIGHTNING.—Dr. J. M. Saunders, Magnolia, South Carolina, writes: "In deciding upon the cause of the death of a person found dead from a supposed stroke of lightning, a physician, in some instances, might have to depend somewhat upon the presence or absence of flaccidity of the body. Beck says: 'The bodies of those killed in this manner are generally, but not always, flaccid.' Recently I examined, 16 hours after death, the body of a black child, nearly nude at the time of its instant death, whose death was undoubtedly caused from lightning, as evidenced from the torn condition of the tenement wherein it sat and the testimony of bystanders. There were no external signs or traces of stroke upon the body discoverable; blood not examined as to fluidity; but the corpse was rigid throughout. Now, if flaccidity be the rule, how long does this condition last? Would it last 16 hours *post mortem?*"

METEOROLOGICAL SUMMARY FOR AUGUST, 1886, STATION, ATLANTA, GA.

| | |
|---|---|
| Mean Temperature, | 76.0 |
| Highest Temperature, August 15th and 16th, | 94.0 |
| Lowest Temperature, August 21st, | 62.0 |
| Monthly Range of Temperature, | 32.0 |
| Greatest Daily Range of Temperature, | 21.0 |
| Least Daily Range of Temperature, | 8.0 |
| Mean Daily Range of Temperature, | 17.0 |
| Prevailing Direction of Wind, | N. W |
| Total movement of wind, | 5,241 miles. |
| Highest Velocity of Wind, Direction and Date, 26 Miles N.W., 1st | |
| Total Precipitation. | 2.36 inches. |
| Number of Days on which .01 in. or more of precipitation fell | 11.0 |
| Number of Foggy Days | .0 |
| " " Clear " | 10.0 |
| " " Fair " | 15.0 |
| " " Cloudy " | .6 |

